# Neurons-derived extracellular vesicles promote neural differentiation of ADSCs: a model to prevent peripheral nerve degeneration

**DOI:** 10.1038/s41598-019-47229-x

**Published:** 2019-08-01

**Authors:** Kelly Cristine Santos Roballo, Juliano Coelho da Silveira, Fabiana Fernandes Bressan, Aline Fernanda de Souza, Vitoria Mattos Pereira, Jorge Eliecer Pinzon Porras, Felipe Augusto Rós, Lidia Hildebrand Pulz, Ricardo de Francisco Strefezzi, Daniele dos Santos Martins, Flavio Vieira Meirelles, Carlos Eduardo Ambrósio

**Affiliations:** 1 Veterinary Medicine Department, Faculty of Animal Sciences and Food Engineering, University of Sao Paulo, Av. Duque de Caxias Norte 225, 13635-900 Pirassununga, SP Brazil; 2 Faculty of Veterinary Medicine and Animal Science, Department of Posgraduation, University National of Columbia, Bogota, Colombia; 3 Experimental and Comparative Pathology Department, Faculty of Veterinary Medicine and Animal Science, University of Sao Paulo, Av. Prof. Orlando Marques de Paiva, 87 - Butantã, 05508-010 São Paulo, SP Brazil

**Keywords:** Mesenchymal stem cells, Stem-cell differentiation

## Abstract

Potential mechanisms involved in neural differentiation of adipocyte derived stem cells (ADSCs) are still unclear. In the present study, extracellular vesicles (EVs) were tested as a potential mechanism involved in the neuronal differentiation of stem cells. In order to address this, ADSCs and neurons (BRC) were established in primary culture and co-culture at three timepoints. Furthermore, we evaluated protein and transcript levels of differentiated ADSCs from the same timepoints, to confirm phenotype change to neuronal linage. Importantly, neuron-derived EVs cargo and EVs originated from co-culture were analyzed and tested in terms of function, such as gene expression and microRNA levels related to the adult neurogenesis process. Ideal neuron-like cells were identified and, therefore, we speculated the *in vivo* function of these cells in acute sciatic nerve injury. Overall, our data demonstrated that ADSCs in indirect contact with neurons differentiated into neuron-like cells. Neuron-derived EVs appear to play an important role in this process carrying SNAP25, miR-132 and miR-9. Additionally, *in vivo* neuron-like cells helped in microenvironment modulation probably preventing peripheral nerve injury degeneration. Consequently, our findings provide new insight of future methods of ADSC induction into neuronal linage to be applied in peripheral nerve (PN) injury.

## Introduction

Interactions between cells can be studied through cell communication mediated by extracellular vesicles (EVs)^1^. These exchanges can persuade physical and phenotypical changes, for example, inducing recipient cells to differentiate into additional cell types^2,3^, thus being extremely useful for cellular therapies applied in nerve injury regeneration^4^.

EVs can be classified according to their size as small extracellular vesicles (30–150 nm) and large extracellular vesicles (100–1000 nm)^5–7^. Small extracellular vesicles (Exo) originate from multivesicular bodies (MVBs) and are characterized by the presence of specific proteins such as HSP70, Tetraspanins CD63 and CD9^8^, and proteins of the endosomal sorting complex required for transport (ESCRT), such as ALIX^9^. Large extracellular vesicles (MVs) originate from cell protrusions and their characterization is still being better studied^10^.

The definitive role of EVs is the transfer of components between cells, for example, transfer of bioactive molecules (i.e. mRNA, miRNA, DNA, proteins and lipids) influencing the target cell in terms of phenotype and function^10^. Transcripts or protein components delivered by these vesicles can activate pluripotent genes or trigger epigenetic modifications^11,12^, playing a key role in intercellular communication, proving useful in regenerative stem cell therapies^11,13–15^.

Adipose-derived stem cells (ADSCs) are a type of mesenchymal stem cell (MSC) able to diffferentiated into mesodermal lineages^16^ and ectodermal lineages as neuron-like cells^17^. In addition, ADSCs can be modulated by other cells to start its differentiation process^18^ and can be an alternative cellular therapy to be used in nerve regeneration and microenvironment modulation after nerve injury^18–21^.

Due to the above context, In the present study, we sought to evaluate: if small EVs were involved in the neural differentiation induction process of ADSCs when in co-culture with neurons (BRC)^18^; if the neuron differentiation followed the same gene pathway followed by neural stem cells; and if differentiated ADSCs (neuron-like cells) were functional *in vivo* as an alternative cell type to be used in nerve injury modulation during the regenerative process.

To answer these questions, we first investigated if neurons could induce the neural differentiation process in ADSCs in a transwell co-culture system^18^, evaluating the microtubule protein beta tubulin III (TUBIII)^22^, as well as the structural protein present in neurons, synaptosomal-associated protein 25 (SNAP25)^23^, a protein required for synapse vesicle formation. Gene expression of *Snap25* and microtubule-associated protein 2 (*Map2*)^24^ and transcript levels of microRNA involved in the neuronal differentiation pathway were quantify and analyzed at three time points.

After we had proof of ADSCs neuronal differentiation capacity in co-culture and timeline progression, we next investigated if EVs were involved in this process. In order to do so we looked at the cellular communication by small EVs^4,25,26^, measuring transcript levels of mRNA and microRNAs related with adult neurogenesis^27^, in co-cultured differentiated ADSCs induced by neurons, and in differentiated ADSCs induced only by neuron-derived EVs, to find the most neuron-like cells, which could be used in the *in vivo* study.

Following this screening, we applied the neuron-like cells to a neuron differentiated ADSCs *in vivo* in a neurotmesis injury. *In vivo* analysis of the injured nerve was performed to validate if these cells could assist in PN regeneration or reduce PN degradation during acute period after an extreme injury.

Our results indicate that, by co-culture, neurons stimulated ADSC differentiation. The EVs appear to play a role in this process carrying SNAP25, miR-9 and miR-132, and have neural induction capacity, though lower than the *transwell* co-culture system. When applied *in vivo*, axon density of a normal sciatic nerve and the group that received the neuron-like cells were similar, suggesting that these cells reduced PN degeneration after injury.

## Results

### Experiment 1- ADSCs differentiation into neuronal lineage and neuron-derived EVs involvement in neural differentiation

#### Neurons promote ADSCs differentiation when in indirect culture

**Figure 1:**
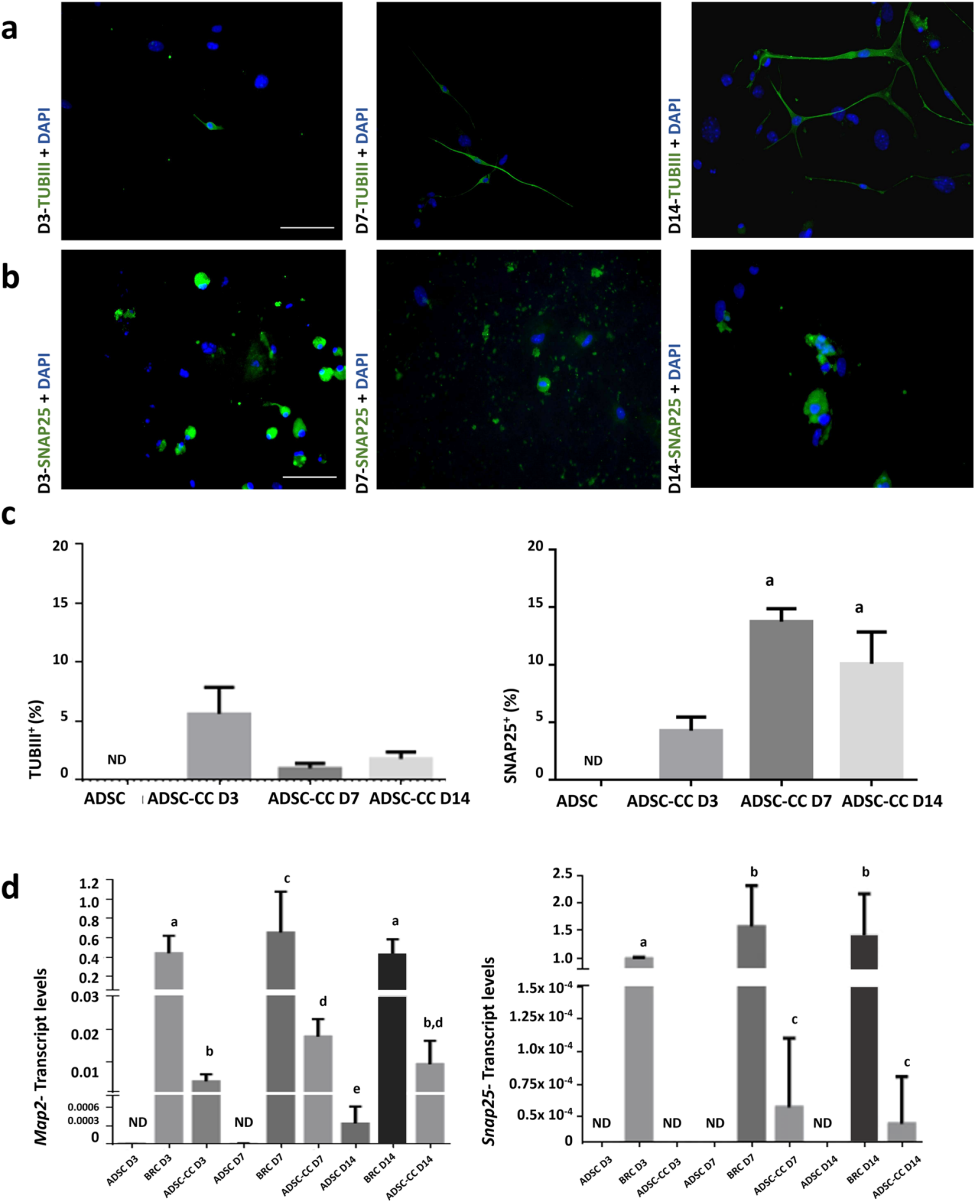
Immunocytochemistry and flow cytometry analysis of SNAP25 and TUBIII, and transcript levels of *Map2* and *Snap25*. (**a**) SNAP25^+^ ADSC-CCs on days 3, 7, and 14, indicating the capacity of BRC to induce the differentiation of ADSC when in co-culture. (**b**) TUBIII^+^ ADSC-CCs on days 3, 7, and 14, indicating the capacity of BRC to induce the differentiation of ADSC when in co-culture. (**c**) Graphs show the percentage of positive cells for TUBIII (left) and for SNAP25 (right) on days 3, 7 and 14. (**d**) Graph on the left shows transcript levels of *Map2*, demonstrating an increase on day 7 compared to day 3 and graph on the right shows transcript levels of *Snap25*, demonstrating an increase on day 7 compared to day 3. TUBIII- beta tubulin III; SNAP25- synaptosomal-associated protein 25. Map2- microtubule-associated protein 2. Different letters indicate p < 0.05. Graph bars correspond to standard error. Bar = 100 µm.

ADSCs were characterized as MSC (Supplementary Fig. S1) and were co-cultured with BRCs, at each timepoint (3, 7 and 14 days). There were co-cultured-ADSCs (ADSC-CCs) positive for both neuronal markers TUB III (Fig. 1a) and SNAP25 (Fig. 1b) at each timepoint.

Similar to immunocytochemistry results, flow cytometry showed ADSC-CC positive for TUBIII at all timepoints (5.64% on D3, 1.0% on D7 and 1.83% on D14) (Fig. 1c), and for SNAP25 at all days with an increase of positive cells from D3 (4.28%) to D7 (13.75%) (Fig. 1c).

We were able to detect *Map2 and SNAP25* mRNAs, with an increase of *Map2* levels in ADSC-CCs on D7 compared to D3 (Fig. 1d) and lower levels of *Map2* on D14 than the other days. Importantly, we were not able to detect *Snap25* in ADSC-CCs on D3; however, we detected some transcripts on D7 and D14 (Fig. 1d).

### Neuron-derived EVs promote neural differentiation of ADSCs

#### Isolation and characterization of small EVs

**Figure 2:**
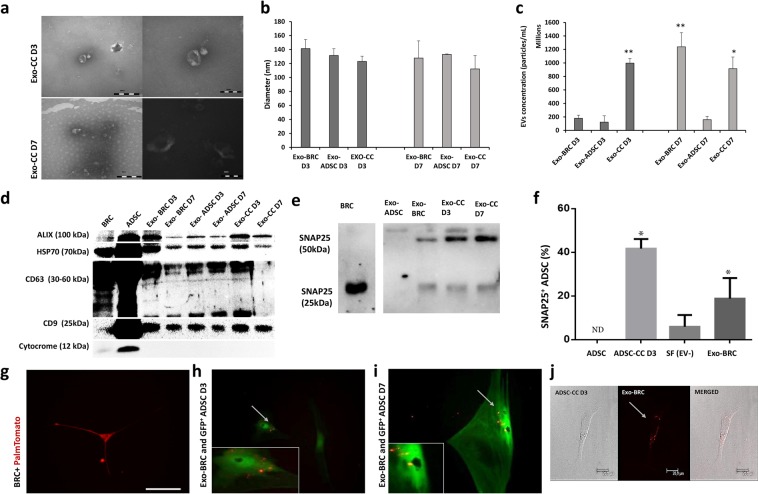
Extracellular vesicles analysis and relationship in ADSC neuronal differentiation - (**a**) Microscopy of transmission of small vesicles from day 3 of co-culture (Exo-CC D3) and day 7 of co-culture (Exo-CC D7). (**b**) Particle diameter (nm) distribution of the isolated particles from control and co-culture (CC) media isolated on day three (D3) and day seven (D7) (Small EVs-Exo) according to nano-tracking analysis. (**c**) Extracellular vesicles concentration according to nano-tracking analysis. (**d**) The western blotting analysis of ALIX, HPS70, CD63, CD9, cytochrome in isolated EVs and control cells (BRC and ADSC). (**e**) The western blotting demonstrating the presence of the SNAP25 protein common in neuronal cells, in small vesicles and abcence in small vesicles from ADSC only. (**f**) Immunocytochemistry quantification demonstrating the presence of the SNAP25 in ADSC (control), ADSC-CC, ADSC cultured with medium without EVs from BRC (Soluble factors-SF), and ASCD cultured Exo-BRC. (**g**) BRC expressing PalmtdTomato reporter (BRC + PalmtdTomato). (**h-i**) Culture of ADSC Exo-BRC + PalmtdTomato after day 3 and 7. (**j**) Co-cultured of ADSC with Exo-BRC + PalmtdTomato after day 3, images demonstrating the presence of EVs + PalmtdTomato together with ADSC. MERGE- merged image showing the extracellular vesicles communication between the cells in the co-culture. Bar = 20 μm. *p ≤ 0.05 and **p ≤ 0.001. The bars correspond to the means and the smaller bars are the standard error of the mean.

The images acquired from transmission electron microscopy confirmed the cup-shape of isolated extracellular vesicles (Fig. 2a). Nanoparticle analysis demonstrated that on isolated Exo the size ranged between 94.5 to 141.4 nm (Fig. 2b). Small EVs concentration at D3: Exo-BRC D3 1.8 97 × 10^8^ particles/mL, Exo-ADSC D3 1.2 × 10^8^ particles/mL, Exo-CC D3 9.97 × 10^8^ particles/mL, and at D7: Exo-BRC D7 1.23 × 10^9^ particles/mL, Exo-ADSC D7 1.5 × 10^8^ particles/mL and Exo-CC D7 9.1 × 10^8^ particles/mL (Fig. 2c). The western blotting analysis demonstrated presence of ALIX, HSP70, CD63 and CD9 in isolated EVs from ADSC and BRC media, and co-cultured media on D3 and D7, and absence of cytochrome in the same samples (Fig. 2d, Supplementary Figs S2 and S3). SNAP25 was found in EVs secreted from co-culture and BRC but not in ADSCs-derived EVs (Fig. 2e and Supplementary Fig. S2). BRC and ADSC cells were used on the western blot as control.

Interestingly, SNAP25 with 25 kDa protein band was detected only in neurons and a very weak band of SNAP25 with 25 kDa was detected only in EVs from neurons and from the co-culture media (Fig. 2e and Supplementary Fig. S2), but strong bands of SNAP25 with 50 kDa and 55 KDa was detected in EVs from neurons and from the co-culture media, but not in EVs from ADSCs (Fig. 2e). Furthermore, after 3 days of culture about 30% of ADSCs cultured with small EVs isolated from BRC were positive for SNAP25 (Fig. 2f), and when in co-culture about 40% of ADSC-CC were SNAP25^+^.

Regarding the EVs tracking analysis, PalmtdTomato reporter was efficiently inserted into EVs from BRC^+PalmtdTomato^ (Exo-BRC) as demonstrated in Fig. 2g. EVs from BRC^+PalmtdTomato^ cultured with ADSCs were observed together with ADSCs but at this point it was not possible to affirm if there was EVs uptake by ADSC or if EVs were in the membrane of ADSCs (Fig. 2h,i). In addition, the transwell co-culture system allowed transfer of EVs from BRC^+PalmtdTomato^ to ADSCs (Fig. 2j).

#### Transcription levels of messenger RNA related to neurogenesis

**Figure 3:**
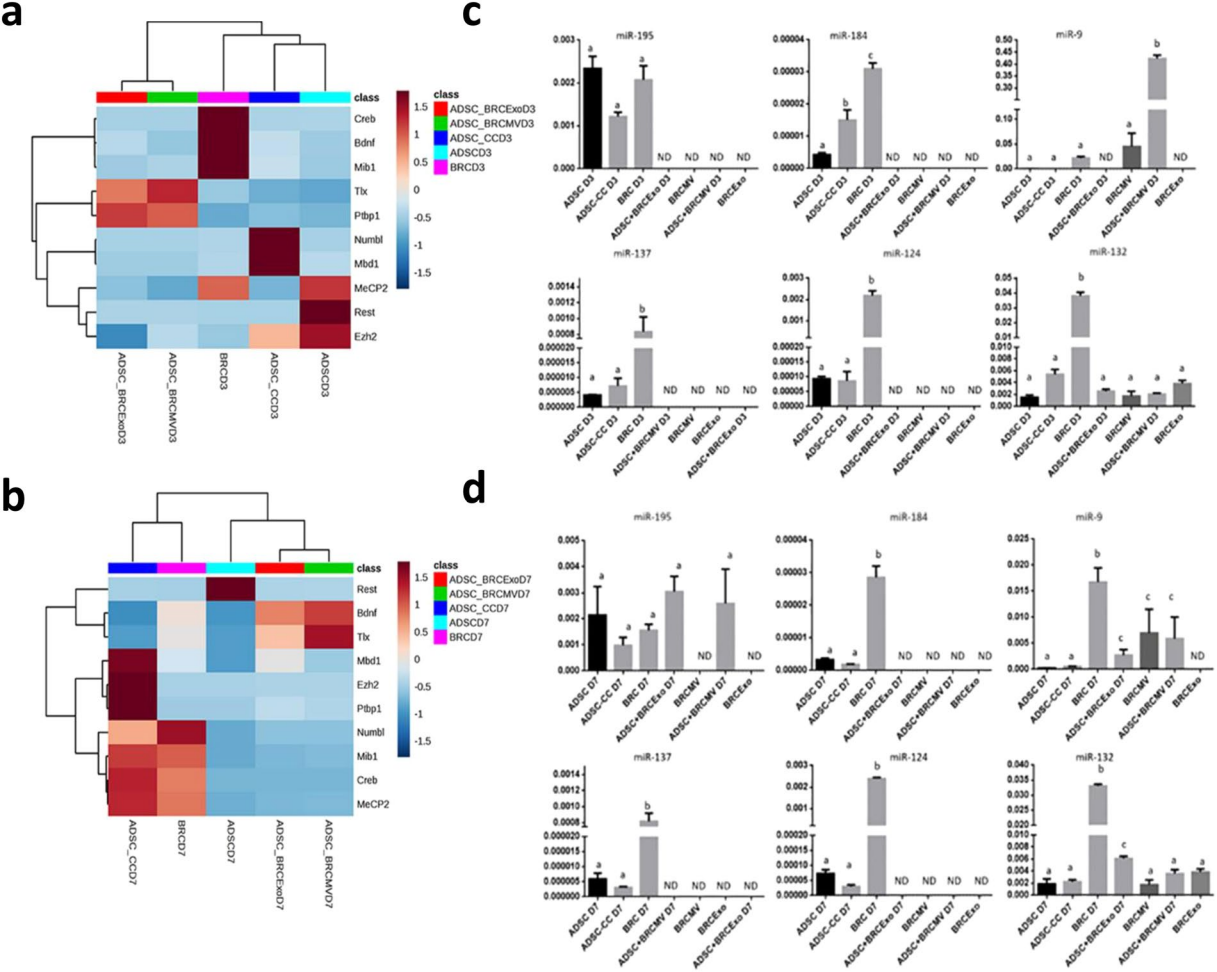
Co-culture and EVs neuronal induction modulating the neurogenic transcript levels. (**a**) Heatmap with hierarchical grouping and analysis of the main genes related with the neurogenesis, showing the differences between ADSC induced or not and brain cells on D3. (**b**) Heatmap with hierarchical grouping and analysis of the main genes related with the neurogenesis, showing the ADSC-CC D7 as the most similar to BRC. (**c**) miRNAs studied in the ADSC induced into neuron-like cells by co-culture or by extracellular vesicles on day 3. There was more expression of miR-184, miR-137, miR-124 and miR-132 in neurons than the other cells types. ADSC-CC showed more expression of miR-184 than the other stem cells, and ADSC + BRCMV D3 showed more expression of miR-9 than the other cells type. ADSC-CC D3 had similar miRNAs levels than ADSC D3, and miR-132 expression was similar in all stem cells. (**d**) miRNAs studied in the ADSC induced into neuron-like cells by co-culture or by extracellular vesicles on day 7. There was more expression of miR-195, miR-184, miR-137, miR-124 and miR-132 in BRC than the other groups. Different letters of the same graph indicate statically difference between the groups. (p ≤ 0.05). ND- no detectable.

On day 3, transcript levels of *Numbl* (p < 0.01) and *Mbd1* (p = 0.0013) were higher in ADSC-CC D3 compared to BRC D3, ADSC + BRCMV D3, and ADSC + BRCExo D3, and were not detected in ADSC D3. *Rest* was higher in ADSC D3 than in BRC D3, ADSC-CC D3, ADSC + BRCMV D3, and ADSC + BRCExo D3 (p = 0.016). *Ezh2* was similarly detected in all cells at this time point (p = 0.1362). Interestingly, *Ptbp1* (p < 0.001) and *Tlx* (p = 0.004) had higher levels in ADSC + BRCMV D3 and ADSC + BRCExo D3 than in cells that did not directly receive EVs from neurons. *MeCP2* was higher in ADSC D3 and BRC D3 than in ADSC-CC D3, ADSC + BRCMV D3, and ADSC + BRCExo D3 (p = 0.0023). The transcript levels of *Bdnf, Creb*, and *Mib1* were higher in BRC D3 than in ADSC D3, ADSC-CC D3, ADSC + BRCMV D3, and ADSC + BRCExo D3 (p < 0.001) (Fig. 3a).

On day 7, the transcript levels for *Mbd1, Ezh2*, and *Ptbp1* increased in ADSC-CC D7 compared to ADSC D7, ADSC + BRCMV D7, and ADSC + BRCExo D7 (p < 0.01). *Tlx* was not identified in ADSC-CC D7 and ADSC D7 but was detected in ADSC + BRCMV D7 and ADSC + BRCExo D7 (p = 0.009). *Rest* had higher expression in ADSC D7 than in BRC D7, ADSC-CC D7, ADSC + BRCMV D7, and ADSC + BRCExo D7 (p = 0.0026). *Numbl, MeCP2, Mib1*, and *Creb* levels were similar in ADSC-CC D7 and BRC D7 compared to ADSC D7, ADSC + BRCMV D7, and ADSC + BRCExo D7 (p < 0.003). *Bdnf was* higher in ADSC + BRCMV D7, ADSC + BRCExo D7, and BRC D7 than in ADSC-CC D7 and ADSC D7 (p = 0.003) Fig. 3b).

#### Transcription levels of micro RNAs related to neurogenesis

On day 3, transcripts for miR-195, miR-184, miR-137, and miR-124 were detectable in ADSC-CC D3, ADSC D3, and BRC D3 and were not detectable in ADSC + BRCMV D3 and ADSC + BRCExo D3 (p < 0.0001). The transcripts for miR-9 were higher in ADSC + BRCMV D3 than in ADSC-CC D3, ADSC D3, and BRC D3 (p < 0.0001). Interestingly mir-9 transcripts were detectable on BRCMV (p < 0.0001) and miR-132 transcripts were detectable in all cells, in higher levels in BRC D3 (p < 0.0001) (Fig. 3c).

On day 7, transcripts for miR-195 levels were detectable in ADSC-CC D7, ADSC D7, BRC D7, ADSC + BRCEXo D7, and ADSC + BRCMV D7 (p = 0.012). The transcripts for miR-184, miR-137, and miR-124 were detected in ADSC-CC D7, ADSC D7, and BRC D7 and were not detectable in ADSC + BRCMV D7 and ADSC + BRCExo D7 (p < 0.0001). Interestingly, miR-9 was higher in BRC D7, ADSC + BRCExo D7, BRCMV, and ADSC + BRCMV D7 than ADSC D7 and ADSC-CC D7, and was not detectable in BRCExo (p = 0.0045). MiR-132 levels were higher in BRC D7 and ADSC + BRCExo D7, and low detected in other samples (p < 0.0001) (Fig. 3d). Interestingly, BRCMV carried miR-9 and miR-132.

### Experiment 2- Neuron-like cells decrease nerve degeneration after peripheral nerve injury

#### *In vivo* imaging

**Figure 4:**
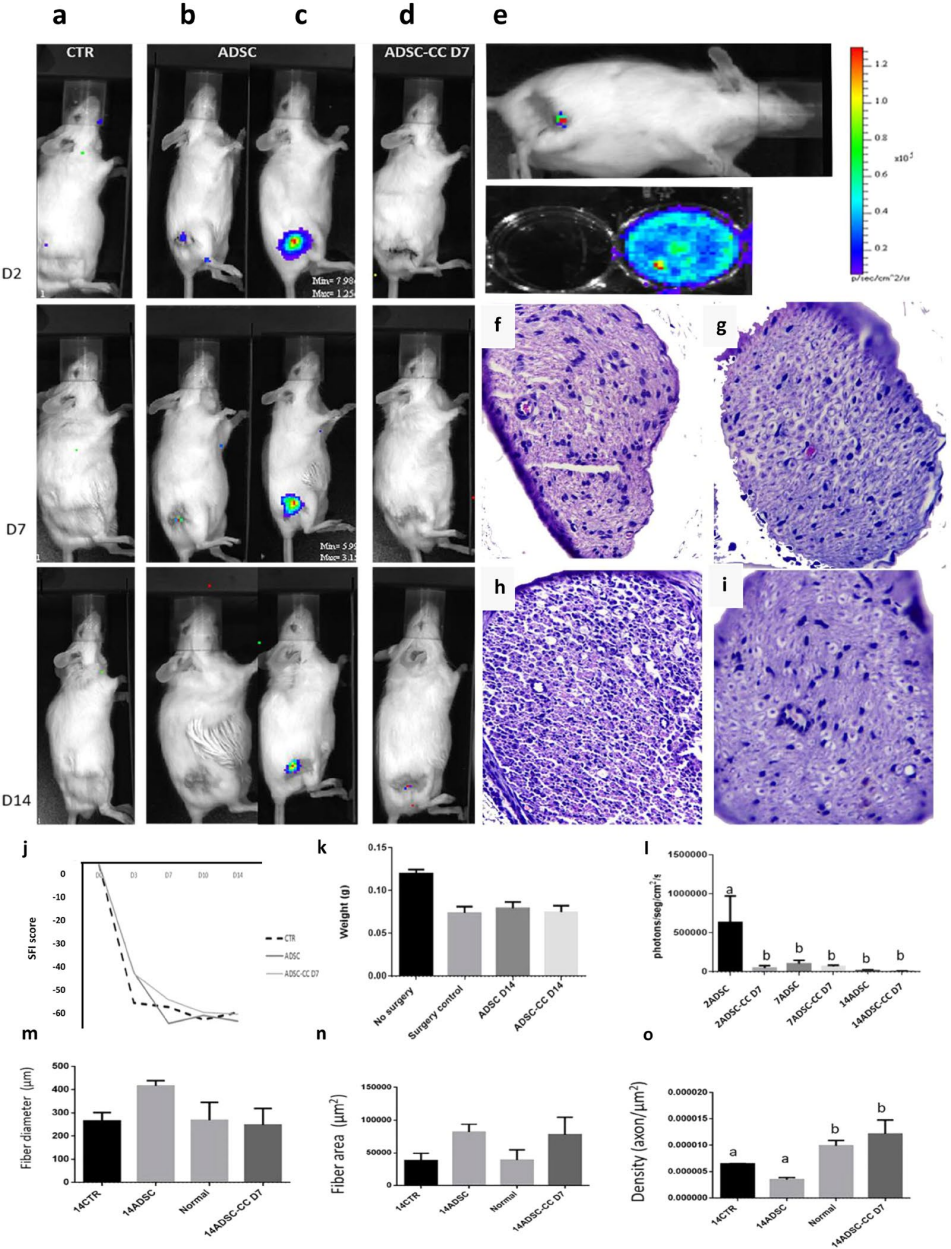
Co-cultured ADSC local environment improvement *in vivo*. (**a**) Example IVIS analysis of the CTR (control group) surgery without stem cell application group. (**b**,**c**) Example IVIS analysis of the surgery with ADSC application group. (**d**) Example IVIS analysis of the surgery with ADSC-CC D7 application group. (**e**) ADSCs-CC D7 were bioluminescent after the differentiation process, and after the application, few bioluminescence was acquired. Signal close to red indicate more bioluminescence and closer to blue indicate few bioluminescence. (**f**) Histology of the sciatic nerve of CTR group (**g**) Histology of the sciatic nerve of ADSC group. (**h**) Histology of the sciatic nerve of no surgery group (Normal). (**i**) Histology of the sciatic nerve of ADSC-CC D7 group. (**j**) Evaluation of sciatic nerve index (SFI) - Differences between SFI in the groups evaluated. Animals that received ADSC-CC D7 showed an improvement in SFI when compared to the other treatments, even though no statistical difference was observed (p ≤ 0.05). (**k**) On the first day of analysis (seventh postoperative day) there were marked differences between the group ADSC-CC D7 and the group ADSC on the wet weight of the gastrocnemius muscle, but over time there were no differences between the experimental groups. (**l**) Graph of IVIS analysis, there was more detectable signal on the ADSC than CTR and ADSC-CC D7. (**m**,**n**) There was no statistical difference between fiber diameter and (**m**) area (**n**) however there was more axon density (**o**) on the ADSC-CC D7 group than ADSC, equal value to the normal nerve without neurotmesis, Different letters within the same graph indicate statistical differences (p ≤ 0.05). The bars correspond to the means and the smaller bars are the standard error of the mean.

It was possible to follow ADSCs Luciferin/GFP+ without co-culture (control cell) on all analyzed days in the IVIS. However, it was not possible to track ADSC-CC D7 Luciferin/GFP+ (Fig. 4a–e,l).

#### Sciatic Functional Index and Gastrocnemius weight analysis

The animals treated with ADSC-CC D7 Luciferin/GFP+ had better SFI scores when compared with the other treatments; however, no statistical difference was noted (p < 0.05) (Fig. 4j). ADSC Luciferin/GFP+ group and ADSC-CC D7 Luciferin/GFP+ presented −41.41 (STDEV) and 41.57 (STDEV) SFI score, respectively, on D3, while the control group presented −52.75 (STDEV) SFI score. On D7 post-surgery, the ADSC-CC D7 group showed −51.58 (STDEV) SFI score, while the ADSC group presented −60.66 (STDEV) SFI score and the control group showed −54.43 (STDEV) SFI score. On D10, the ADSC-CC D7 group still showed a better SFI score than the other groups with −56.39 (STDEV) in comparison with the ADSC group, which presented 57.61 (STDEV) and the control group, −59.12 (STDEV) SFI score (Fig. 4g). Additionally, on D14, gastrocnemius wet weight was similar among all the groups (p < 0.05) (Fig. 4k).

#### Sciatic nerve histomorphometry

Fourteen days after neurotmesis, considered acute window (Fig. 4f–i,m–o), sciatic nerve diameter (Fig. 4m) and nerve fiber area (Fig. 4o) were similar between all analyzed animals and groups (Fig. 4m). Axon density was compared between all groups, there was significant difference between 14ADSC-CC D7 and 14CTR and 14ADSC (p = 0.0043) and resembled the amount found in an uninjured nerve (Normal) (p = 0.0043) (Fig. 4o).

## Discussion

In the presented work, we emphasized the importance of looking at the neuron-derived EVs’ capacity to induce neuronal differentiation in ADSCs. Our results demonstrated that there were more differentiated ADSC-CCs at D7, and this process appears to be mediated by neuron secretions. Importantly, SNAP25, miR-9, and miR-132 were found in neuron-derived EVs, which were taken up by ADSC-CCs, as shown by the EV^+PalmtdTomato^ system. Lastly, we analyzed the *in vivo* proprieties of differentiated ADSCs. The ADSC-CCs D7 applied *in vivo* improved limb functionality after neurotmesis.

In accordance with our findings, recent studies using chemical compounds demonstrated similar expression of neural markers, such as *Map2* and SNAP25 in stem cells differentiated into neuronal lineages^24,28,29^. Regarding the extracellular vesicles findings, the neuron-secreted EVs were involved in ADSCs neuronal differentiation as recently pointed out by Takeda *et al*.^30^.

A novel aspect of this current work is the delivery of SNAP25 by neuron-secreted EVs, which might play a role in neural differentiation. The SNAP25 protein normally weighs 25 kDa, but our findings demonstrated the presence of 50 kDa and 55 kDa protein bands in EVs, which is described in other studies as probably a protein complex^31^.

Another very interesting finding of our study was the confirmation of a crosstalk between neuron-derived EVs and ADSCs by the presence of neuron-secreted EVs^+PalmtdTomato^ within ADSC-CCs. In fact, studies have confirmed that EV-mediated communication is a dynamic and multidirectional mechanism of cell communication, mediating the delivery of functional components^12,13^.

In the context of the transcripts related to adult neurogenesis, in ADSCs co-cultured with BRCs, levels of *Numbl* and *Mbd1* were higher in ADSC-CCs D3 than in all of the other induced ADSCs, which is an indication of neurogenesis as it can be observed in NSCs^28,32^. Importantly, *Mbd1*, which helps to maintain the potentiality of neural stem cells by restricting the differentiation genes and initiating the activation of these genes^33^, was higher in ADSC-CCs D3 and could be an indication of activation of a similar adult neurogenesis pathway^33^. Additionally, ADSC-CCs D7 presented higher levels of *Ezh2*, a gene responsible for the differentiation pathway of NSCs^34^, than ADSC-CCs D3, which indicates an important transcript change in these cells after a certain period of co-culture under neuron induction. Furthermore, *Mib1*, responsible for the neural differentiation process^35^, and *Creb*, involved with neuron maturation and function^36^, were most highly expressed in ADSC-CCs D7, suggestive of neuron maturation of these co-cultured cells.

When the same transcripts were measured in the ADSCs induced by neuron-derived EVs, other transcript profiles were observed.

*Ptbp1* is an RNA regulatory protein and assists in the control of alternative splicing during the process of neural differentiation in progenitor stem cells^37^, controls synapse-related protein genes^38^, and is a target gene of miR-124^39^ and levels of *Bdnf*, which is a neurotrophin that regulates the entire neuronal environment, aiding in the process of neuronal maturation present in neurogenesis^40^ were higher in the induced ADSCs by EVs; which could be an indication that the neural differentiation process was being activated.

Importantly, *Rest* was in low levels in both cells, which indicates that such cells are possibly involved in the process of neurogenesis. *Rest*^41^ and the *CoRest/*MeCP2^42^ complex regulate genes related to quiescence and proliferation prior to the onset of differentiation of NSCs into mature neurons^43^, and when *Rest* is silenced, there are higher rates of reprogrammed fibroblasts for neural differentiation^41^. It is known that *Rest* inhibition is partially modulated by up-regulation of miR-9 and miR-124^41^, but this inhibitory pathway was not observed in the induced ADSCs.

Neuron-secreted EVs appear to play a role in this phenotype change from mesenchymal stem cell to neuron-*like* cells. MiR-9 is highly detected in the brain^44,45^ and its high levels can reduce *Tlx* expression, improving the differentiation of NSCs^46^. In this study, the ADSCs which received neuron-secreted EVs, had high levels of miR-9 and *Tlx* on D3 and miR-9 was detected in the MVs from the neurons, suggesting the transfer of this miRNA. Additionally, miR-132 was highly abundant in all induced ADSCs and presented in all neuron-secreted EVs.

Therapies for the regeneration of peripheral nerve have mostly been evaluated using mesenchymal stem cells under or already differentiated in neuron-like cells^47^, similar to this study, but a highlight of our study was that probably there was a delay of the Wallerian degeneration process during the acute period after an extreme nerve injury by using the neuron-like cells developed in this study. Several studies have shown that even after surgery, full recovery of the peripheral nerve is not a reality^48,49^, even when using cells or allografts^50^ in an effort to delay the degenerative process as an alternative to gain time and improve recovery after surgery.

In conclusion, neurons are able to induce the neural differentiation of ADSCs; confirmed by ADSC-CCs phenotypic changes, as well as the expression of neuronal transcripts. Furthermore, we were able to demonstrate the transfer of neuron-derived EVs to ADSCs using an EV-reporter construct, and the delivery of SNAP25, miR-9, and miR-132, suggesting that cell-secreted vesicles from neurons were related to the neural differentiation of ADSCs. Lastly, the strategy to reduce the process of degeneration using differentiated MSC as the cell type developed in this study can be useful when an increased time during pre-surgery is required due to problems such as infection at the site of injury or non-vascularization, or when the total section of the peripheral nerve is necessary. The use of pre-differentiated cells in neuron-like cells may be an alternative in these cases, since it appears that they aid in improving the function of the affected limb after injury.

## Methods

### Ethics statements

The study procedures were performed in accordance with the Guide for the Care and Use of Laboratory Animals of the National Institutes of Health, and all procedures involving animals were conducted in accordance with the Committee of Ethics of the Faculty of Animal Science and Food Engineer -FZEA - University of Sao Paulo, Pirassununga, SP, Brazil. In addition, all the experimental protocols performed in this study were approved by the Bioethical Committee of the Faculty of Animal Science and Food Engineer -FZEA - University of Sao Paulo, Pirassununga, SP, Brazil, composed of community members and experts in animal care and ethics (animal ethics approval number: 7347240915).

### Experiment 1- ADSCs differentiation into neuronal lineage and neuron-derived EVs involvement in neural differentiation

#### Neurons promote ADSC differentiation when in indirect contact

Neurons and mesenchymal stem cell isolation and culture. We used the FVB mouse strain from the Hemocenter of Ribeirao Preto’s vivarium, Ribeirao Preto, SP, Brazil to collect ADSCs and neurons (BRC) (N = 10, 2 to 3-month old). Cells were isolated and primary cultured was performed. In addition, ADSC was characterized as mesenchymal stem cell by colony unit forming, flow cytometry for mesenchymal markers, and neural differentiation of the ADSCs to prove stem capacity before conducting experiments. For more details: Supplementary Information.

Indirect contact culture using transwell co-culture system. The neurons (10^5^ BRC/mL) and ADSCs (10^4^ ADSC/mL) were co-cultured using the transwell (400 nm, ThinCertsTM, Greiner Bio-One, Kremsmünster, Austria) technique described previously^18^. Cells were co-cultured for 3 (D3), 7 (D7), and 14 (D14) days (time of cell induction).

Immunocytochemistry and flow cytometry analysis. In order to determine the change in phenotype, we performed immunocytochemistry and flow cytometry of SNAP25 and TUBIII (N = 7). For immunocytochemistry, quantification of the percentage of positive cells for SNAP25 and TUBIII markers was obtained according to the literature^51^. For more details: Supplementary Information.

Transduction with eGFP and sorting. Lentiviral production with eGFP was performed by lipofection of 293FT cells (Invitrogen, Carlsbad, California, USA). The ADSCs were cultured in 60 mm petri dishes until attaining 60% confluence, followed by 3 mL incubation with 6 μg/mL of virions particles, and then sorted by flow cytometry using FACSAria Cell Sorter supported by DiVa V.6.1.2 software (BD Biosciences, San Jose, CA, USA) using filters adjusted to the light emission of 525 nm for FITC.

Messenger RNA and micro RNA extraction, reverse transcription of messenger or micro RNA and RT-qPCR analysis. To analyze genes and pathways related to the neurogenesis (Supplementary information Tables 1 and 2) messenger RNA and micro RNAs quantification was performed. For these procedures, three biological and two or more technical replicates were used. For more details: Supplementary Information. Total RNA was isolated from all differentiated ADSCs, control cells (ADSC and BRC), and EVs using TRIzol LS (Life Technologies, Carlsbad, California, USA), following the manufacturer’s protocol. RNA quality was determined by Nanodrop (Thermo Fisher Scientific, Carlsbad, California, USA) based on 260 and 280-ratio analysis.

In summary, for mRNA cDNA synthesis was performed using the High Capacity Reverse Transcription Kit (Applied Biosystems, Carlsbad, CA, USA), following the manufacturer’s protocol (genes and primers are listed in Supplementary Information, Tables 1 and 2). For microRNA, miScript PCR System (num. 218193, Qiagen, Hilden, Germany) was used according to the manufacturer’s protocol, with a total of 100 ng of total RNA (microRNAs listed, and primers listed, Tables 3 and 4 of Supplementary Information).

#### Neuron-derived EVs promote neural differentiation of ADSC

Isolation and characterization of small EVs. From control media and co-culture media of the three periods (D3, D7, D14) small EVs were isolated using ExoQuick, their shape was analyzed using transmitted microscopy and ALIX, CD63, CD9, HSP70, cytochrome, calnexin, COX IV was also analyzed by western blot^52–55^. For nanoparticle quantification we used NanoSight NS300 (Malvern Instruments, Malvern, UK) with a laser of 405 nm and NanoSight NTA software v2.3 to evaluate the EVs from D3 and D7. EVs were diluted in DPBS (1:100). Five videos of 30 seconds were recorded with 14-camera level at 37 °C for each sample after calibration with 100 nm of beads (Malvern Instruments, Malvern, UK) to ensure equipment accuracy as indicated by the manufacturer. The concentration was defined from the average of the recorded movies. For more details: Supplementary Information.

PalmtdTomato labeled EVs. We used the plasmid vector kindly provided by Dr. Charles P. Lai (National Tsing Hua University, Hsinchu, Taiwan)^56^, containing the tandem dimer Tomato (tdTomato) fused at NH2-termini with a palmitoylation signal (PalmtdTomato) to label EVs. To summarize, neurons were transduced with a lentivirus vector encoding PalmtdTomato for 24 hours, then the media was changed and EV experiments were performed.

Small EVs funtion analysis. To confirm EVs communication from neurons to ADSCs, we performed two culture conditions: (i) ADSC cultured with small extracellular vesicles (Exo) ^+PalmtdTomato^ from BRC^+PalmtdTomato^, Exos were isolated using ExoQuick and added to ADSC GFP+ or ADSC GFP-; (ii) co-culture of ADSC GFP- and BRC^+PalmtdTomato^ using transwell system.

Additionally, the number of ADCS positive for SNAP25 from these two culture conditions and the presence or absence of SNAP25 in the small EVs were measured.

EVs messenger RNA and micro RNA extraction, reverse transcription of messenger or micro RNA and RT-qPCR analysis. To analyze genes and pathways related to the neurogenesis (suplemmentary information  Tables 1 and 2) transcript level analysis of ADSC cultured with neuron-derived small extracellular vesicles (ADSC + BRCExo) and ADSC cultured with neuron-derived large vesicles (ADSC + BRCMV) from D3 and D7 was performed. We selected D3 because it was the suggested day when ADSCs were starting the neuronal differentiation and D7 because it was the day with a higher rate of differentiated ADSC-CC and according to our transcript analyzes with ADSC similar to BRC. Transcript levels of specific microRNAs in control cells (ADSC and BRC) and in BRC EVs was used as baseline.

### Experiment 2- Neuron-like cells decrease nerve degeneration after peripheral nerve injury

#### *In vivo* study design

Twenty-eight healthy 2 to 3-month old, irrespective of gender, FVB mice, weighing 15–25 g, and three FVB luciferin/GFP+ ADSC donors were provided by the animal research area of Hemocenter of Ribeirao Preto, Sao Paulo, Brazil, for this experiment. They were kept in the vivarium of the Faculty of Animal Science and Food Engineer -FZEA - University of Sao Paulo, Pirassununga, SP, Brazil, in standard conditions to reduce suffering and distress: 12-hour light/dark cycle, 20–22 °C; feed (balanced lab block made of high quality ingredients) and water supplied ad libitum; and environmental enrichment in the form of paper roles to reduce the stress. Conditions were the same for all experimental groups, both before and after surgery procedures.

The animals were divided into three groups: Control group (CTR) – surgery procedure and neurotmesis; ADSC – surgery procedure, neurotmesi with, and ADSC application; and ADSC-CC D7 – surgery procedure, neurotmesis with ADSC-CC D7 application. Analysis was performed after 14 days of the surgery procedure, during acute period.

#### Surgery procedures and cell delivery

The animals were anesthetized using ketamine Chlorhydrate (90–150 mg/Kg), xylazine (7.5–16 mg/Kg), and tramadol (20–40 mg/Kg) administered intraperitoneally. While the animals were under anesthesia, an incision in the skin was made, and the gluteal musculature was moved in order to reveal the right sciatic nerve. The sciatic nerve was exposed and cut (neurotmesis), and 5 µL of PBS alone or of 5 × 10^5^ cells (ADSC or ADSC-CC D7) suspended in 5 µL of PBS were added to the injury site. The muscle and skin incisions were then closed, and the animals were kept under supervision until recovery. The post-operative procedures and method of euthanasia are described in the Supplementary Material.

#### *In vivo* imaging

On days 2, 7, and 14 after surgery, animals were imaged using *In Vivo* Imaging - IVIS® Lumina XRMS (IVIS). The scans were performed using a bioluminescence protocol with 5 minutes of exposure and image analysis was performed. For more details: Supplementary Material.

#### Sciatic functional index and gastrocnemius weight analysis

We followed the Bain *et al*.’s^57^ protocol, with some adaptations for sciatic functional index (SFI), the method was performed on days 3, 7, 10, and 14. For gastrocnemius weight analysis we followed Wang *et al*.’s^58^ protocol.

#### Sciatic nerve histomorphometry

The sciatic nerve of each animal was isolated and processed. The axon size, axon quantification per micrometer square, and nerve diameter was quantified and compared between the groups. For more details: Supplementary Information.

#### Statistical analysis used in all experiments and techniques

The statistical analyses were performed using GraphPad Prism 6 (GraphPAd Software, Inc. San Diego, CA). Analysis of variance (ANOVA) with p ≤ 0.05 was used, followed by Tukey’s test when P-values were significant. All data are presented as mean ± SEM.

## Supplementary information

Supplementary material
