# Supplementary material for: Neurons-derived extracellular vesicles promote neural differentiation of ADSCs: a model to prevent peripheral nerve degeneration
Source: Sci Rep. 2019 Aug 1;9:11213. doi: 10.1038/s41598-019-47229-x (PMC6671995; doi:10.1038/s41598-019-47229-x)
Supplement: Supplementary file 1 — Supplementary material [file 41598_2019_47229_MOESM1_ESM.doc]

Neurons-derived extracellular vesicles promote neural differentiation of ADSCs: a model to prevent peripheral nerve degeneration

Kelly Cristine Santos Roballo, Juliano Coelho da Silveira, Fabiana Fernandes Bressan, Aline Fernanda de Souza, Vitoria Mattos Pereira, Jorge Eliecer Pinzon Porras, Felipe Augusto Rós, Lidia Hildebrand Pulz, Ricardo de Francisco Strefezzi, Daniele Santos Martins, Flavio Vieira Meirelles, Carlos Eduardo Ambrósio.

**Supplementary Figures**

**Supplementary Fig. S1**

**
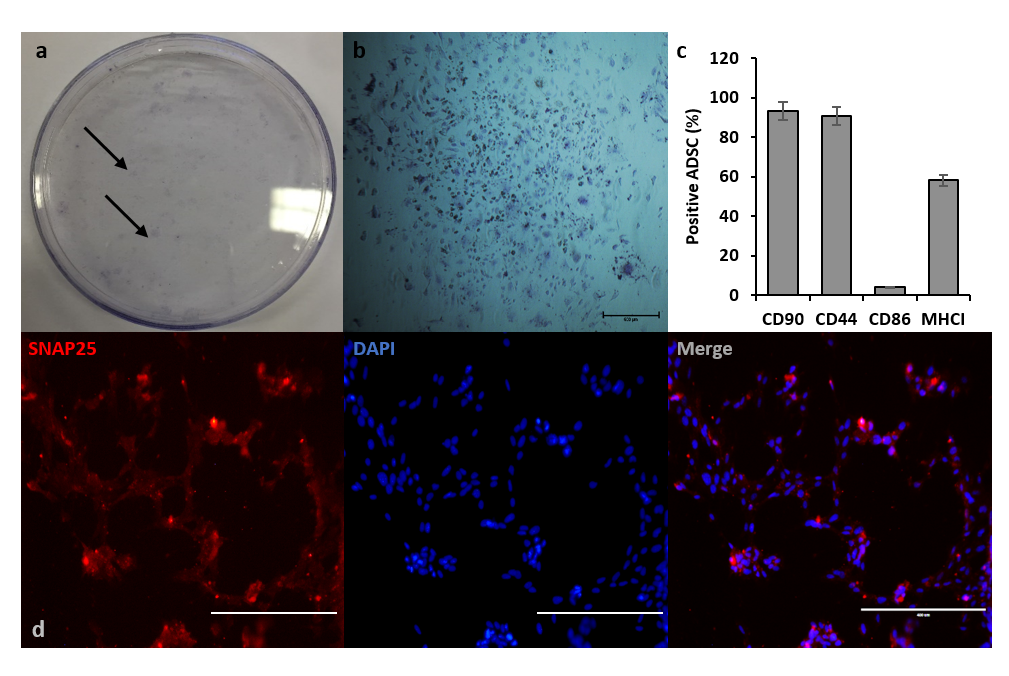
**

**Supplementary Fig. S1. ADSC characterization.** (**a**) Colonies of ADSC, arrows indicate the colonies (**b**) Microscopy image of ADSC colony; scale bar= 600 µm. (**c**) Mesenchymal markers on ADSC. (**d**) SNAP25+ ADSC after chemical neural differentiation, scale bar=400µm.

**Supplementary Fig. S2**

**
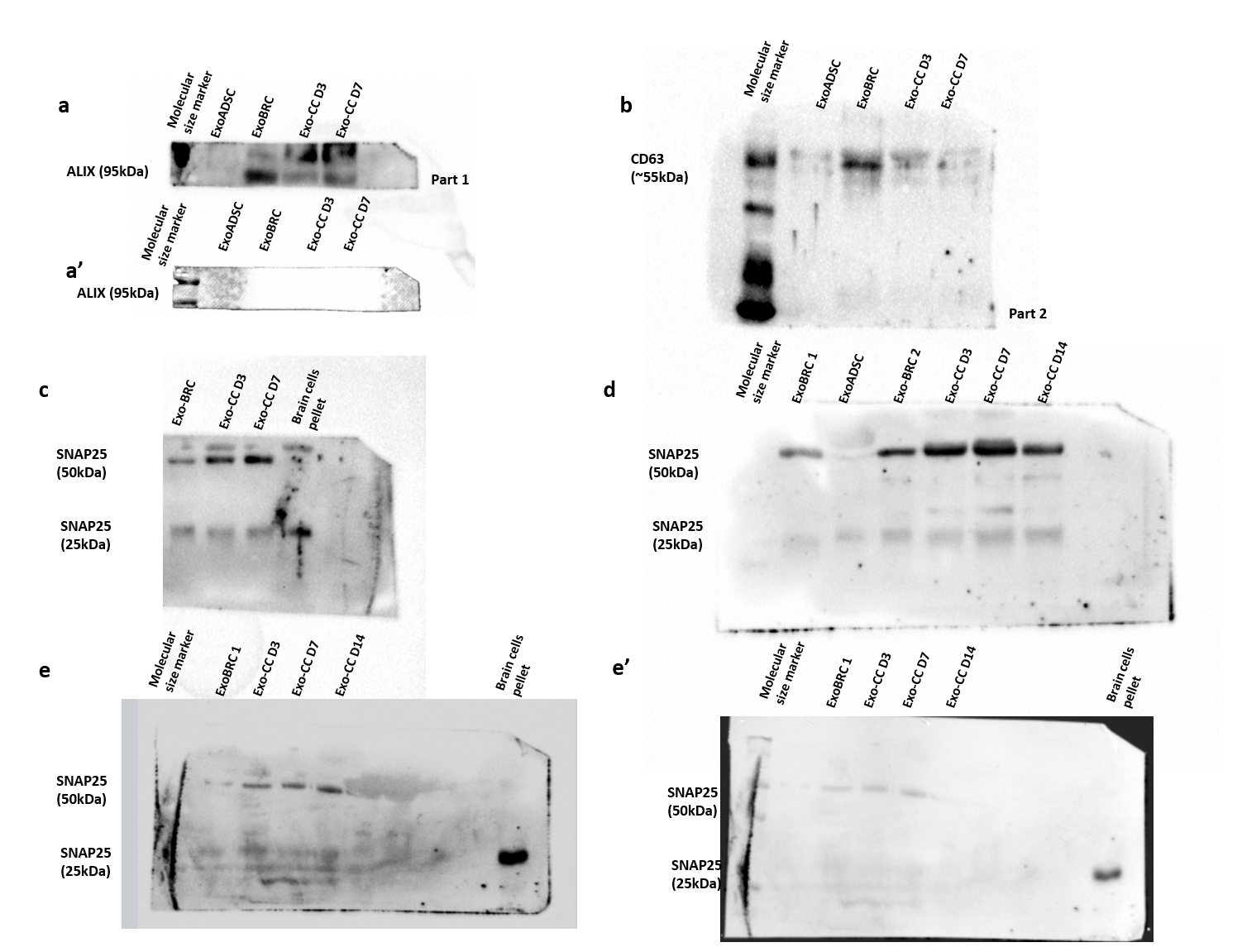
**

**Supplementary Fig. S2.** Full images of western blot membranes. (**a** - **a’**) Western blot analysis of ALIX in Exos from ADSC medium, BRC medium and co-culture medium (part 1). (**b**) Western blot analysis of CD63 in Exos from ADSC medium, BRC medium and co-culture media (part 2 from the gel where ALIX was marked). (**c**) Western blot analysis of SNAP25 in EVs from BRC media, co-culture media and brain cells pellet (control). (**d**) Western blot analysis of SNAP25 in EVs from ADSC medium, BRC media, co-culture media and brain cells pellet (control). (**e** - **e’**) Western blot analysis of SNAP25 in EVs from BRC media, co-culture media and brain cells pellet.

**Supplementary Fig. S3**


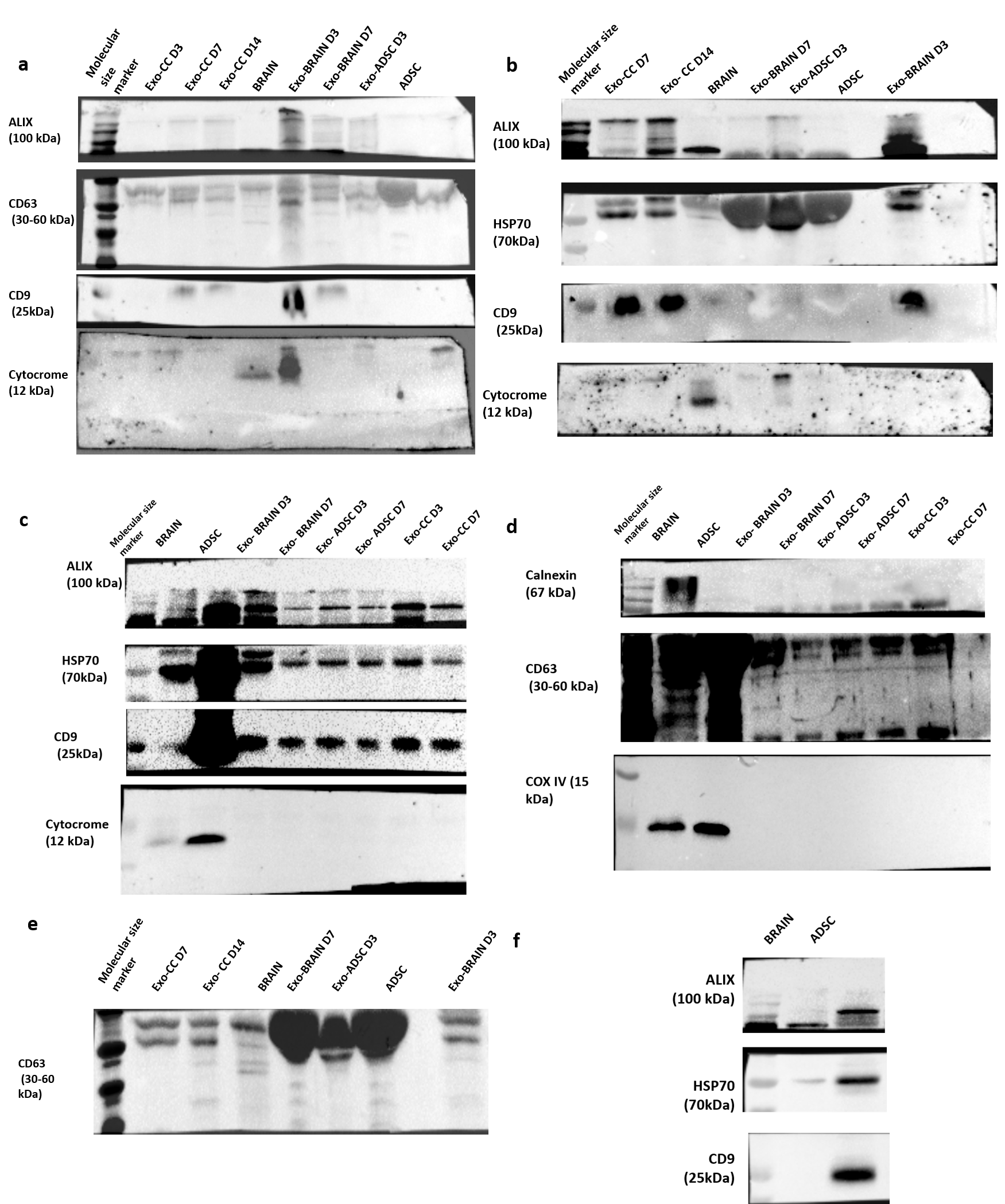


**Supplementary Fig. S3.** Full images of western blot membranes. (**a**) Western blot analysis of ALIX, CD63, CD9, and Cytocrome in Exos from ADSC medium, BRC medium and co-culture medium, and control cells (BRC and ADSC). (**b**) Western blot analysis of ALIX, HSP70, CD9, and Cytocrome in Exos from ADSC medium, BRC medium and co-culture medium, and control cells (BRC and ADSC). (**c**) Western blot analysis of ALIX, HSP70, CD9, and Cytocrome in Exos from ADSC medium, BRC medium and co-culture medium, and control cells (BRC and ADSC). (**d**) Western blot analysis of calnexin, CD63, COX IV in Exos from ADSC medium, BRC medium and co-culture medium, and control cells (BRC and ADSC). (**e**) Western blot analysis of CD63 in Exos from ADSC medium, BRC medium and co-culture medium, and control cells (BRC and ADSC). (**f**) Western blot analysis of ALIX, HSP70, CD9 in control cells (BRC and ADSC).

**Supplementary Material and Methods**

**Neurons and ADSC isolation**

We used the FVB mouse strain to collect adipose and brain cells (BRC) (N=10, 2 to 3-month old, males and females) To summarize, adipose tissue samples were mechanically and enzymatically digested at 37°C for 2 hours using collagenase type I (17100017, Gibco® life technologies, Gaithersburg, MD, EUA), and the brain cells were mechanically isolated for 15 minutes. Following isolation, cells were centrifuged for 5 min at 1,500rpm, and the pellet was suspended in DMEM/High Glucose (Dulbecco's Modified Eagle Medium high glucose, Gibco® life technologies, Gaithersburg, MD, EUA) supplemented with 10% KnockOut™ Serum Replacement (10828028, Thermo Fisher Scientific, Waltham, MA, USA) and penicillin/streptomycin (1%; PAA, Sigma-Aldrich, St. Louis, MO, USA). 10^5^ cells/ml were plated in 6 wells plate

**ADSC characterization**

We performed colony unit forming, flow cytometry for mesenchymal markers CD90 (CD90-anti-goat (igG) sc6071, Santa Cruz Biotechnology, Dallas, Texas, USA), CD44 (MCA 1082GA, AbD, Serotek, Raleigh, NC, USA), CD86 (NB100-77815, BD, San Jose, CA, USA) and MHCI (MCA1086GA. AbD, Serotek, Raleigh, NC, USA), and neuronal differentiation of the ADSCs to prove stem capacity before conducting experiments.

**Immunocytochemistry**

In order to determine the change in phenotype, we performed immunocytochemistry repeated in 7 biological replicates and 3 techniques. Briefly, each protein of interest was evaluated in triplicates obtained from the cells of 7 animals. Cultured cells were fixed in 4% paraformaldehyde (PFA) pH 7.2- 7.4 for 12 minutes and washed in DPBS (Dulbecco’s Phosphate Buffered Saline, Sigma-Aldrich, St. Louis, MO, USA) prior to incubation with 1% Triton X-100 in DPBS for 30 minutes. Samples were then blocked at room temperature with 1% BSA (Bovine Serum Albumin, Sigma-Aldrich, St. Louis, MO, USA) in DPBS for 1 hour. Following incubation of the primary antibody (1:500 rabbit polyclonal anti-beta tubulin III, ab18207, Abcam, Cambridge, MA, EUA; 1:400 goat polyclonal anti-Snap25, SC-7538, Santa Cruz Biotechnology, Dallas, Texas, USA) overnight at 4°C, cells were washed and incubated with secondary antibody (1:1000 goat anti-rabbit FITC, ab6717, Abcam, Cambridge, MA, EUA; 1:1000 goat anti-mouse FITC, ab97249, Abcam, Cambridge, MA, EUA; 1:500 Alexa Fluor 594 donkey anti-goat, A11058, Invitrogen, Thermo Fisher Scientific, Waltham, MA, USA) for 1 hour at room temperature the next day. Negative controls were performed by omitting the primary antibodies. We also performed nuclei staining with Hoechst (Trihydrochloride, Trihydrate, 33342, Invitrogen, Thermo Fisher Scientific, Waltham, MA, USA) and DAPI (DAPI 4′,6-Diamidine-2′-phenylindole dihydrochloride, Sigma Aldrich, St. Louis, MO, USA). All data were acquired using a light microscope and fluorescence microscopy (AXIOVISION 4.7.1, Carl Zeiss, Oberkochen, Germany) at 10x and 20x magnification.

**Quantification of immunopositive cells**

The percentage of positive cells for TUBIII and SNAP25 markers was obtained by counting positive and negative cells attributed to the markers in five randomly chosen fields until an amount of 500 counted cells was reached. This procedure was repeated in 7 biological replicates with 3 techniques for each marker using the program Image J.

**Flow cytometry**

Cell suspension of ADSC-CC at 5×10^5^ concentration from each co-culture period (N=7) was fixed in 4% PFA pH 7.2- 7.4 for 12 minutes, washed with FACS buffer, and then incubated in 1% BSA for 1 hour. After incubation, cells were placed in primary antibody (1:500 rabbit polyclonal anti-beta tubulin III, ab18207, Abcam, Cambridge, MA, EUA; 1:400 goat polyclonal anti-Snap25, SC-7538, Santa Cruz Biotechnology, Dallas, Texas, USA) for 1 hour at room temperature, washed 3 times for 5 minutes with DPBS, incubated with secondary antibody (1:1000 goat anti-rabbit FITC, ab6717, Abcam, Cambridge, MA, EUA; 1:1000 goat anti-mouse FITC, ab97249, Abcam, Cambridge, MA, EUA; 1:500 Alexa Fluor 594 donkey anti-goat, A11058, Invitrogen, Thermo Fisher Scientific, Waltham, MA, USA) for 30 minutes at room temperate, and washed 3 times for 5 minutes with DPBS. Cells were analyzed by flow cytometry using FACSAria Cell Sorter supported by DiVa V.6.1.2 software (BD Biosciences, San Jose, CA, USA), using filters adjusted to the light emission of 525nm for FITC, 488nm for Alexa flour 488, and 470nm for DAPI fluorochromes. Flow cytometry data were analyzed using FCS Express 5 Flow Research Edition.

**Reverse transcription of messenger RNA and PCR**

The cDNA synthesis was performed using the High Capacity Reverse Transcription Kit (Applied Biosystems, Carlsbad, CA, USA), following the manufacturer’s protocol, with a total of 1000ng per sample. Polymerase chain reactions were performed using ABI-7500 with SYBR Green Master Mix Kit (Applied Biosystems, Carlsbad, CA, USA) for the selected neuronal genes (Table 1 and Table 2).

The program used for the RT-qPCR was: 1 cycle of 95°C for 10 minutes, 45 cycles of 95°C for 15 seconds, 1 cycle of 58°C for 1 minute, 1 cycle of 95°C for 15 seconds, 1 cycle of 60 °C for 1 minute, and 1 cycle 95°C for 15 seconds. Melting curve analysis was then performed to verify the amplification of specific products. Transcript levels were determined by RT-qPCR and the cycle threshold (Ct) values of the target gene were normalized by GAPDH (endogenous gene) Ct values for each sample. Following normalization, fold change was calculated using the 2^–ΔCT^ equation method. Three biological and two technical replicates were used.

**Table 1. Genes involved in the adult neurogenesis27, 29**

| ***Gene*** | ***Function*** |
| --- | --- |
| ***Numbl*** | NS self-renew |
| ***Mbd1*** | Transcript regulation, NSC differentiation |
| ***Rest*** | Silencing of neuronal genes |
| ***Tlx*** | Essential for self-renew and NSC proliferation |
| ***Ezh2*** | NSC fate |
| ***MeCP2*** | Related with pre-mature neurons |
| ***Ptbp1*** | Transition of NSC to differentiation |
| ***Bdnf*** | Axonal development |
| ***Mib1*** | Neuronal differentiation stimuli |
| ***Creb*** | Mature neuron function and synapse regulation |

**Table 2. Genes used on the RT-q PCR**

| ***Gene*** | ***Forward primer (5′→3′)*** | ***Reverse primer (5′→3′)*** | ***Reference*** |
| --- | --- | --- | --- |
| ***Gapdh*** | TGACATCAAGAAGGTGGTGAAGC | CCCTGTTGCTGTAGCCGTATTC | 59 |
| ***Map2*** | AGCCGCAACGCCAATGGATT | TTTGTTCTGAGGCTGGCGAT | 59 |
| ***Snap25*** | AGAATCGCCAGATTGACAGG | ACCACTTCCCAGCATCTTTG | 60 |
| ***Numbl*** | GGGGACAAGAAGAAAGCCGA | TCCCTTCTCTCCAGGGGATG | NM_010950.2 |
| ***Rest*** | TCGCCACTTGGTGAATGTGT | CAAAGTCTGCACGGTGCTTC | NM_011263.2 |
| ***Ezh2*** | TCCATGCAACACCCAACACA | CCTTAGCTCCCTCCAGATGC | NM_001146689.1 |
| ***Ptbp1*** | TCGACTTCTCCAAGCTCACC | ACGGAGAGGCTGACATTATGC | NM_001077363.2 |
| ***Bdnf*** | TCTTTTCCGAGGTTCGGCTC | CTCACCTGGTGGAACCGGAG | NM_001285416.1 |
| ***Mib1*** | TATACTGCCTTGCACCTGGC | GCACGGACCAAAAGCCTAC | NM_144860.2 |
| ***Creb*** | ACAAGCGAAACCAACAAACCAT | CGTCTGAGGCTGGTTCATGT | NM_001025432.1 |
| ***Mbd1*** | CGCCAGTGATCACGGAGATT | GCTTTTCAGCCACAAAAGAATGT | NM_013594.2 |
| ***Tlx*** | GCACAACCAATAGCCACCTG | AAATGCGGCTTGTTGATCCG | NM_152229.2 |
| ***MeCP2*** | CTGTAGACCAGCTCCAACAGG | CTGCAGAATGGTGGCTGAA | NM_010788.4 |

**Western Blotting analysis**

Proteins were isolated from the EVs and brain cells using RIPA buffer (Sigma-Aldrich, St. Louis, MO, USA) according to the manufacturer’s protocol. Proteins were resolved in 8% polyacrylamide SDS-PAGE for 2 hours in 100V (Bio-Rad, Hercules, CA, USA) gel. Next, proteins were transferred to a nitrocellulose membrane (Biotrace NT, Pall Life Sciences, Pensacolla, FL, USA) for 30 minutes at 25V using the semi-dry apparatus. After transfer membranes were incubated with blocking solution (5% BSA in TBST - Tris Buffered Saline with Tween® 20) for 1 hour at room temperature, the membranes were incubated overnight at 4°C in primary antibody (1:500 goat polyclonal anti-Alix, SC-49267; 1:500 rabbit polyclonal IgG CD63, SC-15363; 1:800 goat polyclonal anti-Snap25, SC-376713, Santa Cruz Biotechnology, Dallas, Texas, USA). The membranes were washed 3 times for 5 minutes with 1X TBST, followed by incubation of secondary antibody (1:3000 donkey anti-goat IgG HRP-linked, SC-2020, Santa Cruz Biotechnology, Dallas, Texas, USA;1:3000 goat anti-rabbit IgG HRP-linked, #7074, Cell Signaling Technology, Danvers, MA, USA) for 1 hour at room temperature. The membranes were washed 3 times for 5 minutes with 1X TBST, and then ECL Plus Prime Western Blotting Detection System solution (Amersham™, Buckinghamshire, UK) was added for color reaction. The reactions were measured by band visualization through ChemiDoc MP Image System (Bio-Rad, Hercules, CA, USA). We conducted this procedure in 3 animals from each group.

**Transmission electron microscopy**

We followed Thery et al. (2006)’s protocol with some modifications. Isolated EVs from the co-cultured media were fixed in 2.5% glutaraldehyde, 0.1M cacodylate, and 4% paraformaldehyde pH 7.2-7.4 for 2 hours at room temperature, followed by 100,000g ultracentrifugation for 80 minutes, then washed with 4mL of ultrapure water, and followed by a second ultracentrifugation at the same time and speed. The pellet was suspended in 100µL of ultrapure water and added to a copper grid coated with pioloform for five minutes. The excess liquid was withdrawn with wet filter paper, 2% of uranyl acetate drops were added to the grid for three minutes, and then the grid was read in the transmission electron microscopy FEI at 200kV, Tecnai20, LAB6.

***In vivo* imaging**

On days 2, 7, and 14 after surgery, animals were imaged using *In Vivo* Imaging - IVIS® Lumina XRMS. To do so, mice were anesthetized in a holding chamber with 4% isoflurane in compressed air, injected with D-luciferin (VivoGlo™ Luciferin, *In Vivo* Grade, P1043, Promega, Madison, Wisconsin, USA), following the manufacturer’s protocol (150mg/Kg, IP), and moved to the scanner after 15 minutes, maintaining anesthesia with 2.5% isoflurane. The scans were performed using a bioluminescence protocol with 5 minutes of exposure and image analysis was performed.

**Sciatic nerve histomorphometry**

The sciatic nerve of each animal and group was isolated and processed. After 14 days, fixed in 4% PFA for 24 hours, processed on paraffin blocking (Histosec®, Merck), and the sections were stained with H&E for the measurements. The axon size, axon quantification per square, and nerve diameter was quantified and compared between the groups. The H&E slides were analyzed under 4X, 40X and 100X magnification on a LEICA DM500 microscope (Wetzlar, Germany).

**Table 3.** MiRNAs involved in the adult neurogenesis

| **mmu-miRNA** | **Target gene** |
| --- | --- |
| **miR-195a-5p** | *Mbd1* |
| **miR-184-3p** | *Numbl* |
| **miR-9-5p** | *Tlx, Rest* |
| **miR-137-3p** | *Ezh2, Mib1* |
| **miR-124-3p** | *Ptbp1* |
| **miR-132-3p** | *MeCP2, Creb* |

**Table 4.** Homolog between bta-miRNA and mmu-miRNA

| **bta-miRNA** | **Sequence** | **mmu-miRNA** | **Sequence** | **Homolog (%)** |
| --- | --- | --- | --- | --- |
| **miR-9-5p** | UCUUUGGUUAUCUAGCUGUAUG | **miR-9-5p** | UCUUUGGUUAUCUAGCUGUAUGA | 95.65 |
| **miR-124a** | UAAGGCACGCGGUGAAUGCCAAG | **miR-124-3p** | UAAGGCACGCGGUGAAUGCC | 86.95 |
| **miR-132** | UAACAGUCUACAGCCAUGGUCG | **miR-132-3p** | UAACAGUCUACAGCCAUGGUCG | 100 |
| **miR-137** | UUAUUGCUUAAGAAUACGCGUAG | **miR-137-3p** | UUAUUGCUUAAGAAUACGCGUAG | 100 |
| **miR-184** | UGGACGGAGAACUGAUAAGGGU | **miR-184-3p** | UGGACGGAGAACUGAUAAGGGU | 100 |
| **miR-195** | UAGCAGCACAGAAAUAUUGGCA | **miR-195a-5p** | UAGCAGCACAGAAAUAUUGGC | 95.45 |

**References**

1. Oh, S. I. *et al*. Efficient reprogramming of mouse fibroblasts to neuronal cells including dopaminergic neurons. *Sci. World Journal*. **2014**: 957548 (2014).
2. Han, H. *et al*. Effects of chronic fluoride exposure on object recognition memory and mRNA expression of SNARE complex in hippocampus of male mice. *Biol. Trace. Elem. Res.* **158**, 58 (2014).
